# A finite element analysis on different bone cement forms and injection volumes injected into lumbar vertebral body in percutaneous kyphoplasty

**DOI:** 10.1186/s12891-022-05522-3

**Published:** 2022-06-28

**Authors:** Xun Zhang, Tiantian Chen, Fanchao Meng, Shiwen Li, Gongping Xu, Jinglong Yan, Wei Zhao

**Affiliations:** 1 Department of Orthopedics, The First Affiliated Hospital of Harbin Medical University, Harbin, 150001 China; 2 Department of Orthopedics, The Second Affiliated Hospital of Harbin Medical University, Harbin, 150081 China

**Keywords:** Percutaneous kyphoplasty, Bone cement, Finite element analysis, Biomechanics

## Abstract

**Background:**

To investigate the stress changes between different bone cement forms and injection volumes in adjacent vertebrae after percutaneous kyphoplasty (PKP) by establishing a three-dimensional finite element model of osteoporosis.

**Methods:**

A male healthy volunteer was selected. CT of scans L1 to L3 vertebrae were imported into Mimics 21.0 software.The vertebral model of osteoporosiswas established based on previous literature reference. The models were divided into three groups: unilateral, bilateral integration and bilateral separation groups, with each group injecting 2 ml, 4,ml and 6 ml of bone cement, respectively. In all models, a vertical compressive load of 500 N, anterior flexion/posterior extension, left/right bending, and left/right rotation were applied with a moment of 7.5 N/m, of which 85% was applied to the anterior mid-column and 15% to the posterior column. The stress changes between adjacent vertebrae under different conditions were calculated.

**Results:**

After percutaneous kyphoplasty was applied to the L2 vertebral body, some differences can be found between the effects of different cement injection volumes and cement morphology on adjacent structures. There was no major difference between the groups when the bone cement injection volume was 2 ml. When the amount of bone cement injected was 4 ml, the bone cement morphology of the bilateral integration group (BIG) produced less stress between adjacent vertebral bodies. The minimum stress was 14.95 MPa in the L3 vertebral body in posterior extension. Whereas the stress levels on adjacent intervertebral structures, BIG shaped bone cement shows some superiority. In addition, the adjacent vertebrae and intervertebral structures are subjected to less stress during left and right rotation.

**Conclusions:**

The present finite element study suggested that bilateral integration bone cement is a suitable form of cement injection, and when the injection volume is 4 ml, reduces stress on adjacent segments by approximately 15% while maintaining the stability of the injected vertebral body.

## Introduction

Osteoporosis increases the incidence of fractures because of the increased brittleness of the bone [1]. The risk of osteoporosis increases with age, and osteoporotic vertebral compression fractures (OVCF) have been reported to occur every 22 s in people aged > 50 years worldwide [2]. It is well known that osteoporotic vertebral fractures cause persistent pain, kyphotic deformity,and limitation of motion, which affects the quality of life and mortality [3]. OVCFs can generally be treated conservatively [4], but they also carry a range of complications. Conservative treatment is usually not suitable for elderly patients because of the high risk of decubitus ulcers, crushing pneumonia,and venous thromboembolism of the lower extremities [5]. And the risk of failure through open surgery using endosseous metal implants is also higher due to the lower quality of bone in osteoporosis [6]. Galibert introduced vertebroplasty, in which medical bone cement (polymethylmethacrylate) was injected into the fractured vertebral body [7]. Since then, it has been widely used as a minimally invasive procedure for the treatment of osteoporotic vertebral fractures and continues to be so today, as it provides rapid pain relief and allows for early recovery [8]. In recent years, percutaneous vertebroplasty (PVP) and percutaneous kyphoplasty (PKP) have gradually been widely used and have shown remarkable results, especially in the treatment of OVCF. In vertebroplasty, bone cement is injected under high pressure through a percutaneous injection cannula into the medullary space of the collapsed vertebral body. In contrast, in kyphoplasty, the balloon is usually first placed percutaneously into the vertebral body. As it is filled with fluid, the balloon compacts the cancellous bone and creates a cavity. After deflation of the balloon, the cavity formed facilitates controlled placement of the bone cement [9]. Garfinet al. study also highlighted the role of percutaneous kyphoplasty (PKP) in correcting spinal deformities, reducing pain, and maintaining spinal stability [10]. Thus, PKP represents the current frontline treatment for patients with OVCF.

Despite these advantages, some complications are frequently encountered in OVCF treatment, short-term complications such as severe reaction to the bone cement, dural injury during puncture, and compression of the dura and nerve roots in the spinal canal due to bone cement leakage [11]. While distant complications such as adjacent segment fractures also occur occasionally, some studies have reported an increased risk of new compression fractures in adjacent vertebrae after vertebroplasty, with reported incidence rates ranging from 6.2% to 51.9% [12, 13]. Most of these fractures occur in adjacent segments, and changes in vertebral body strength increase the fracture risk, caused by differences in the amount and distribution of cement injected into the vertebral body [14].

Therefore, the purpose of this study was to investigate the stress changes in the adjacent segmental vertebral structures between different cement morphologies and different cement injection volumes during PKP surgery. To investigate the above issues, a three-dimensional finite element (FE) model was used.

## Method

We obtained finite element models of the L1-L3 vertebrae by scanning the vertebrae of healthy male patients, and subsequently selected three female patients and extracted different morphological bone cement models from their CT data. Finally, the different bone cement models were combined with the vertebral body models to obtain the target model for finite element analysis. Under six loading conditions, including anterior flexion, posterior extension, left/right bending, and left/right rotation, the changes in the maximum von Mises stresses on the cortical bone, upper and lower endplates, and annulus fibrosus of the adjacent segmental vertebrae were investigated with different cement injection patterns and injection volumes.Used software Mimics 21.0 (Materialise, Belgium), Geomagic Wrap 2017 (Geomagic, USA), SolidWorks 2018 (Dassault Systemes, USA), ANSYS 19.0 (ANSYS, USA). A CT scanner (GE, USA) was used to collect raw data in DICOM format with a scan slice of 0.625 mm.

### Establishing the finite element model

The L1-L3 finite element model containing the complete three vertebrae and each accessory structure was established. The geometric model of the L1-L3 vertebral body was taken from a 64-slice spiral CT image (scan conditions: 140 kV, 200 mA, layer thickness 0.625 mm, no spacing) of a healthy 26-year-old male (90 kg, 185 cm) with no history of spinal injury or osteoporosis, and no radiological evidence of degeneration. The DICOM file obtained from the CT scan was imported into Mimics software for 3D model reconstruction. The reconstructed model is imported into Geomagic Wrap 2017 software in STL format, and all vertebrae are optimized for smooth processing, removing pegs, filling holes, editing contour lines, constructing surfaces and grids, fitting surfaces. The generated geometric model is imported into SolidWorks 2018 software to assemble the vertebral body and create the intervertebral disc, upper and lower endplates, and articular cartilage. For vertebral body cortical bone thickness, some studies used 1 mm or 1.5 mm [15, 16]. In this study, the cortical bone thickness was set to 1.5 mm. the intervertebral disc consisted of the nucleus pulposus and the annulus fibrosus. The area of the nucleus pulposus was set to 50% of the total area of the disc. The generated model was saved as a SLDPRT format.

### Establishing the bone cement model

**Fig. 1:**
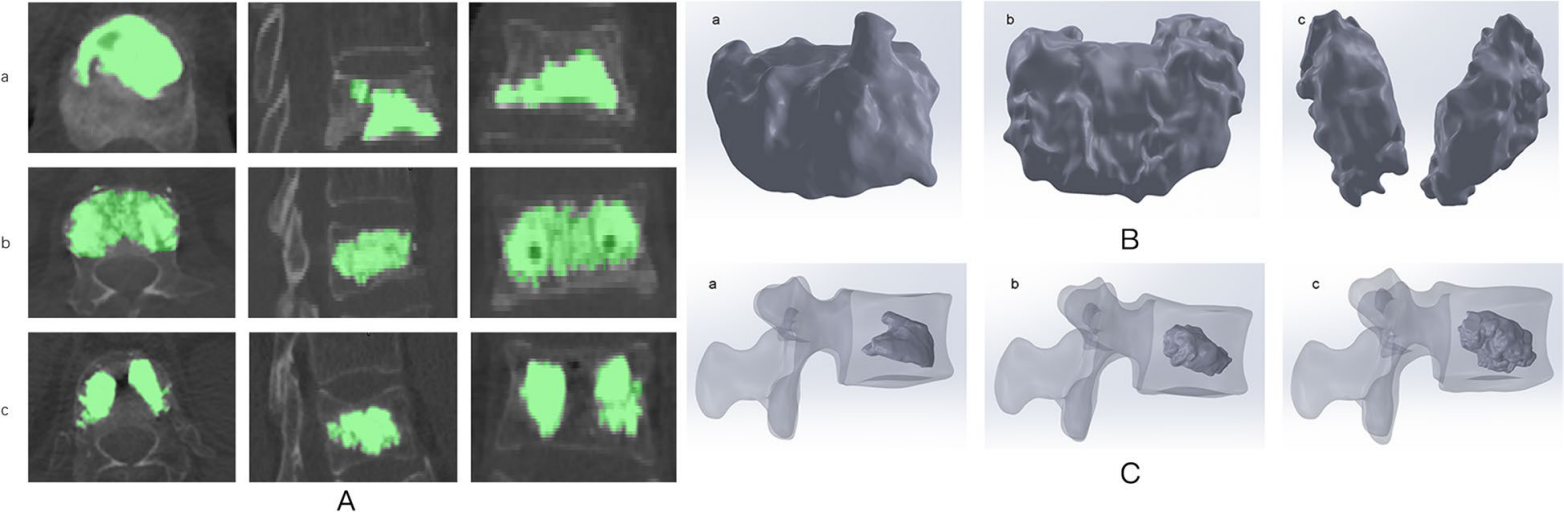
**A** The computed tomography data of bone cement **B** Three different forms of bone cement **C** Bone cement was placed into the L2 pine bone model; a unilateral group; b Bilateral integration group; c Bilateral separation group

Three female patients with vertebral compression fractures were collected, one of whom had a unilateral bone cement injection and the other two patients had a bilateral bone cement injection, which formed two different distribution patterns ("separated" and " integrated "). After obtaining consent from the above three patients, the diseased vertebra was scanned using 64-slice spiral CT. Scanning conditions: 140 kV, 200 mA, layer thickness 0.625 mm, no interval. The bone cement was reconstructed using Mimics software, and three bone cement morphologies were obtained (Fig. 1). The bone cement models were imported into Geomagic Wrap 2017 software in STL format, and the bone cement models were optimized to obtain the final bone cement morphology (Fig. 1). The generated three bone cement models were imported into SolidWorks 2018, and the 2 ml, 4 ml, and 6 ml bone cement models were created by the scale scaling tool, and the different bone cement models were saved into a SLDPRT format.

### Establishment of the complete model

**Fig. 2:**
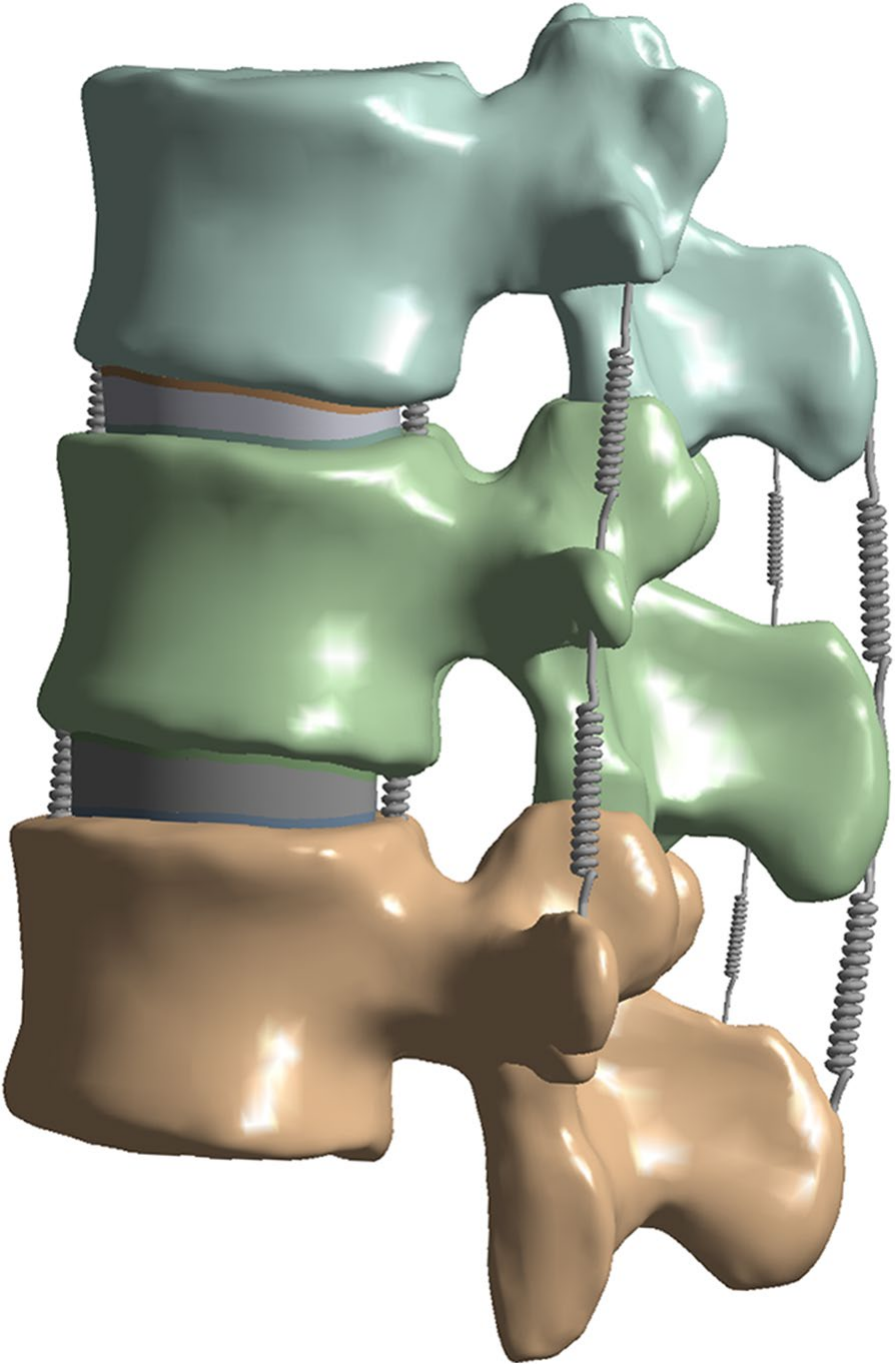
The L1-L3 finite element models

The bone cement and L2 cancellous bone were imported into Solidwork software in part format, and the bone cement with different milliliters and different puncture methods were assembled into the center of the L2 cancellous bone using the assembly command (Fig. 1). A three-dimensional model of the cement-reinforced L2 cancellous bone was obtained by removing excess bone with the Boolean function. The reinforced L2 cancellous bone was assembled with other vertebral structures to get nine complete vertebral models. Finite element analysis was performed using ANSYS 19.0 software. The complete 3D model was imported into ANSYS software, and the additional components included the anterior longitudinal ligament, posterior longitudinal ligament, interspinous ligament, supraspinous ligament, ligament flava, and intertransverse process ligament (Fig. 2). Also, in agreement with previous studies [17, 18], respective characteristics of the various ligaments of the cortical bone, cancellous bone, upper and lower endplates, annulus fibrosus, nucleus pulposus, articular cartilage, and vertebral bodies were defined (Table 1). The element types of anterior longitudinal ligament, posterior longitudinal ligament, interspinous ligament, supraspinous ligament, ligament flava, and intertransverse process ligaments allow for tensile deformation without compressive behavior. The endplates, cortical bone, cancellous, cement, and intervertebral discs (nucleus pulposus and annulus fibrosus) were divided into 2 mm meshes, while the articular cartilage was split into 0.5 mm meshes [19]. The software itself generated the meshes and nodes. Each model endplate was well connected to the vertebral body, endplate to the intervertebral disc, and cortical bone to cancellous bone. The articular cartilage was bound to the superior vertebral body and connected to the inferior vertebral body in a non-separable manner.

**Table 1:** Material parameters of bone and ligaments

Parts	Elastic modulus (MPa)	Poisson’s ratio	Sectional area (mm^2^)
Normal cortical bone	12,000	0.3	
Osteoporotic cortical bone	8040	0.3	
Normal cancellous bone	132	0.2	
Osteoporotic cancellous bone	34	0.2	
Bone cement	3000	0.41	
Endplate	23.8	0.28	
Nucleus pulposus	8.4	0.48	
Annulus fibrosus	92	0.45	
Cartilage	10	0.4	
Anterior longitudinal ligament	8	0.25	65
Posterior longitudinal ligament	10	0.45	20
Intertransverse ligament	40	0.45	1.8
Interspinous ligament	12	0.45	40
Supraspinous ligament	12	0.45	30
Ligamenta flava	20	0.45	40

### Finite element analysis

Static analysis was performed for all nine models, with the lower edge of the L3 vertebral body being fully restrained. Vertical loads and six different loads such as anterior flexion, posterior extension, left/right bending, and left/right rotation was applied to the L1 vertebral body. In order to simulate the weight of the human upper body, the vertical compressive load was 500 N in all models, and a moment of 7.5 N/m was applied to simulate forward flexion/rear extension, left/right bending, and left/right rotation in daily human life. According to the concept of three columns of the spine, 85% of them are applied to the anterior-middle column and 15% to the posterior column [20, 21]. This study aimed to assess the biomechanical changes in the adjacent vertebral bodies of the injured spine. Therefore von Mises stresses in the L1, L3 cortical bone, upper and lower endplates, and annulus fibrosus were calculated to assess the stress changes on the adjacent vertebral structures between different cement injection volumes and injection methods.

### Rationalities of the models

Based on previously published finite element analyses and biomechanical tests, different loading conditions were applied to the model using ANSYS 19.0 software, including forwarding flexion, back extension, lateral bending, and combined loading [22, 23]. The tested values of this model are in agreement with the published results, and all the tested values are within the range of the values of the previous tests. Therefore, the model in this study is valid for further analysis (Table 2).

**Table 2:** Comparison of range of motion between the current intact model and models from previous studies (°)

**Loading condition**	**Chosen parameters**	**Spinal levels**	
		**L1-L2**	**L2-L3**
3.5 Nm FLX			
Guan et al.	Intervertebral rotation(deg)	2.21 ± 0.70	2.46 ± 1.24
Present study	Intervertebral rotation(deg)	1.67	2.36
3.5 Nm EXT			
Guan et al.	Intervertebral rotation(deg)	1.44 ± 0.76	1.43 ± 0.52
Present study	Intervertebral rotation(deg)	1.96	1.35
3.5 Nm LAT			
Guan et al.	Intervertebral rotation(deg)	2.63 ± 0.68	2.66 ± 1.09
Present study	Intervertebral rotation(deg)	2.48	2.73
7.5 Nm FXT with 1175 N FL			
Dreischarf et al.	Intradiscal pressure(MPa)	1.81 ± 0.1	1.5 ± 0.4
Present study	Intradiscal pressure(MPa)	1.80	1.48
7.5 Nm EXT with 500 N FL			
Dreischarf et al.	Intradiscal pressure(MPa)	0.6 ± 0.4	0.5 ± 0.3
Present study	Intradisscal pressure(MPa)	0.68	0.59
7.8 Nm LAT with 700 N FL			
Dreischarf et al.	Intradiscal pressure(MPa)	0.7 ± 0.3	0.7 ± 0.4
Present study	Intradiscal pressure(MPa)	0.98	1.03

*FLX* flexion, *EXT* extension, *LAT* lateral bending, *FL* follower load

## Results

### Stress changes between adjacent vertebrae

**Fig. 3:**
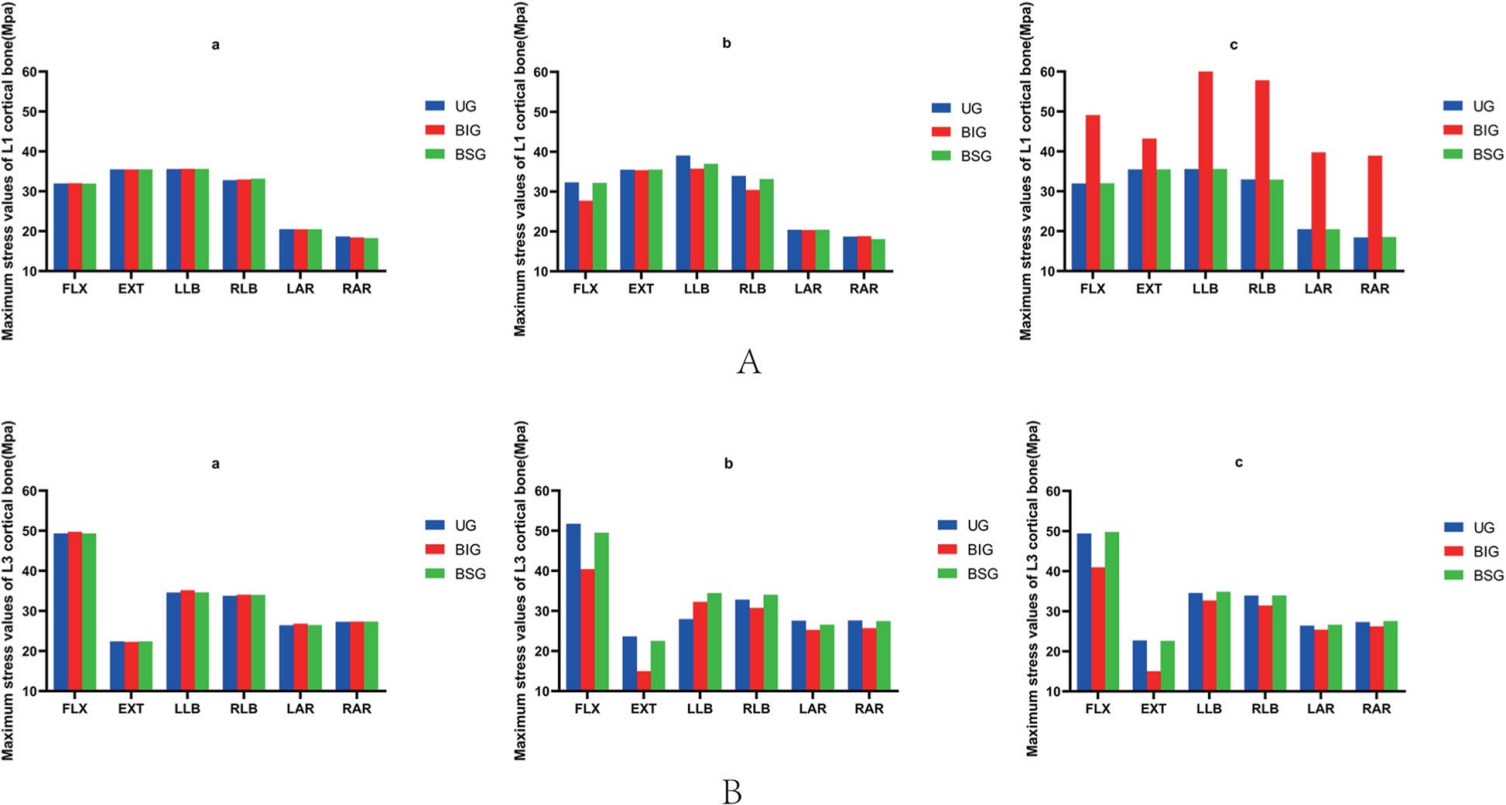
**A** The maximum stress values of L1 cortical bone. **B** The maximum stress values of L3 cortical bone. a 2 ml;b 4 ml;c 6 ml;UG, unilateral group;BIG, Bilateral integration group;BSG, Bilateral separation group

After percutaneous kyphoplasty was applied to the L2 vertebral body, when the bone cement injection volume was 2 ml, there was no major difference in the stresses in the adjacent vertebrae of the three different bone cement forms. When the bone cement injection was 4 ml, the maximum stress on the L1 and L2 vertebrae was less for the BIG bone cement form than for the remaining two bone cement forms. The minimum stress occurs at posterior extension of the L3 vertebral body with a Von Mises stress of 14.96 MPa. When the bone cement injection is 6 ml, the BIG morphology has greater stress in the L1 vertebral body than the remaining two cemented morphologies, but less stress in the L3 vertebral body than the remaining two cemented morphologies. In addition to this, the L3 vertebrae are subjected to greater stress than the L1 vertebrae under all forms of motion (Fig. 3).

### Stress changes between end plates

**Fig. 4:**
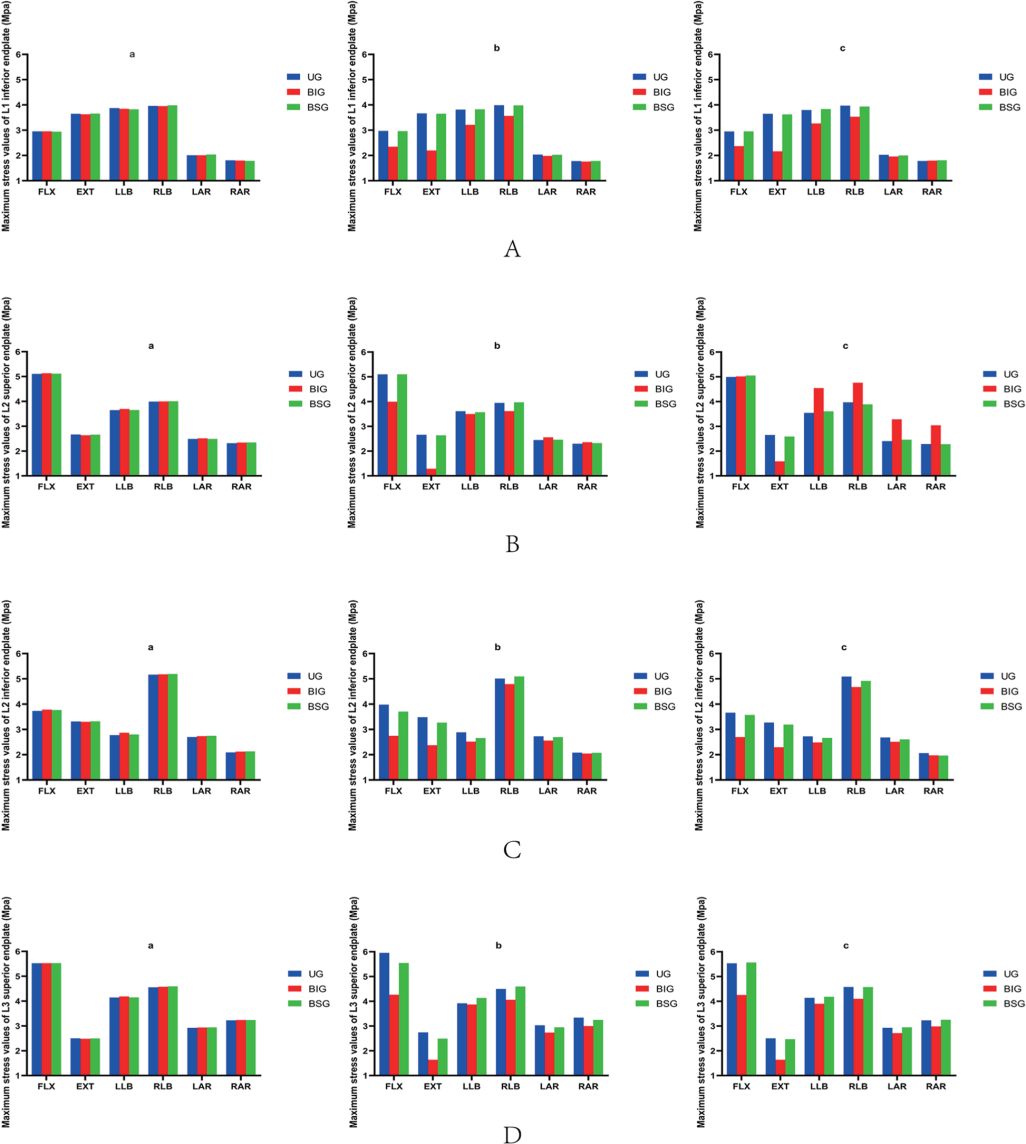
**A** The maximum stress values of L1 inferior endplate. **B** The maximum stress values of L2 superior endplate. **C** The maximum stress values of L2 inferior endplate. **D** The maximum stress values of L3 superior endplate. a 2 ml;b 4 ml;c 6 ml;UG,unilateral group;BIG, Bilateral integration group;BSG, Bilateral separation group

After percutaneous kyphoplasty was applied to the L2 vertebral body, when the bone cement injection volume was 2 ml, there was no major difference in the stresses in the adjacent vertebrae of the three different bone cement forms. When the amount of cement injected was greater than 2 ml, the intervertebral structure was less stressed in the BIG cement form compared with the other two cement forms, and the L2 upper end plate is subjected to the least stress during posterior extension, and the Von Mises stress is 1.29 MPa. Moreover, regardless of the cement morphology, the stress on the upper endplate of L3 is the highest among the adjacent intervertebral structures. In all three cement patterns, the stress on the endplate is greater in anterior flexion and right bending. However, the stress on the endplate was less in right and left rotation than in the other motion patterns (Fig. 4).

### Stress changes between annulus fibrosus

**Fig. 5:**
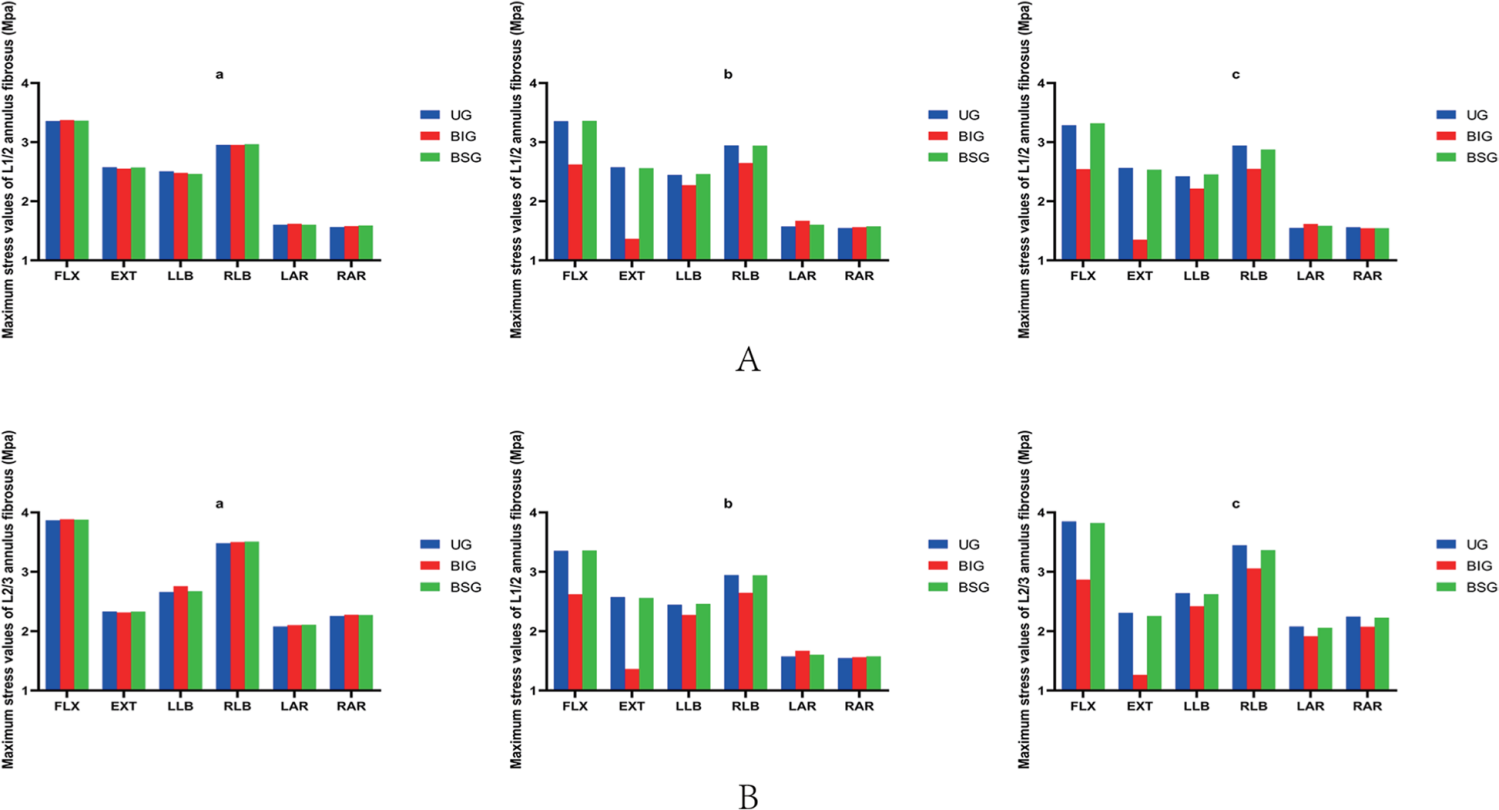
**A** The maximum stress values of L1/2 Annulus fibrosus. **B** The maximum stress values of L2/3 Annulus fibrosus. a 2 ml;b 4 ml;c 6 ml;UG, unilateral group;BIG, Bilateral integration group;BSG, Bilateral separation group

After percutaneous kyphoplasty was applied to the L2 vertebral body, when the bone cement injection volume was 2 ml, there was no major difference in the stresses in the adjacent vertebrae of the three different bone cement forms. When the amount of cement injected was greater than 2 ml, the intervertebral structure was less stressed in the BIG cement form compared with the other two cement forms, and the L23 annulus fibrosus is subjected to the least stress during posterior extension, and the Von Mises stress is 1.26 MPa. Moreover, regardless of the cement morphology, the stress on the L23 annulus fibrosus is the highest among the adjacent intervertebral structures. In all three cement patterns, the stress on the endplate is greater in anterior flexion and right bending. However, the stress on the endplate was less in right and left rotation than in the other motion patterns. (Fig. 5).

**Fig. 6:**
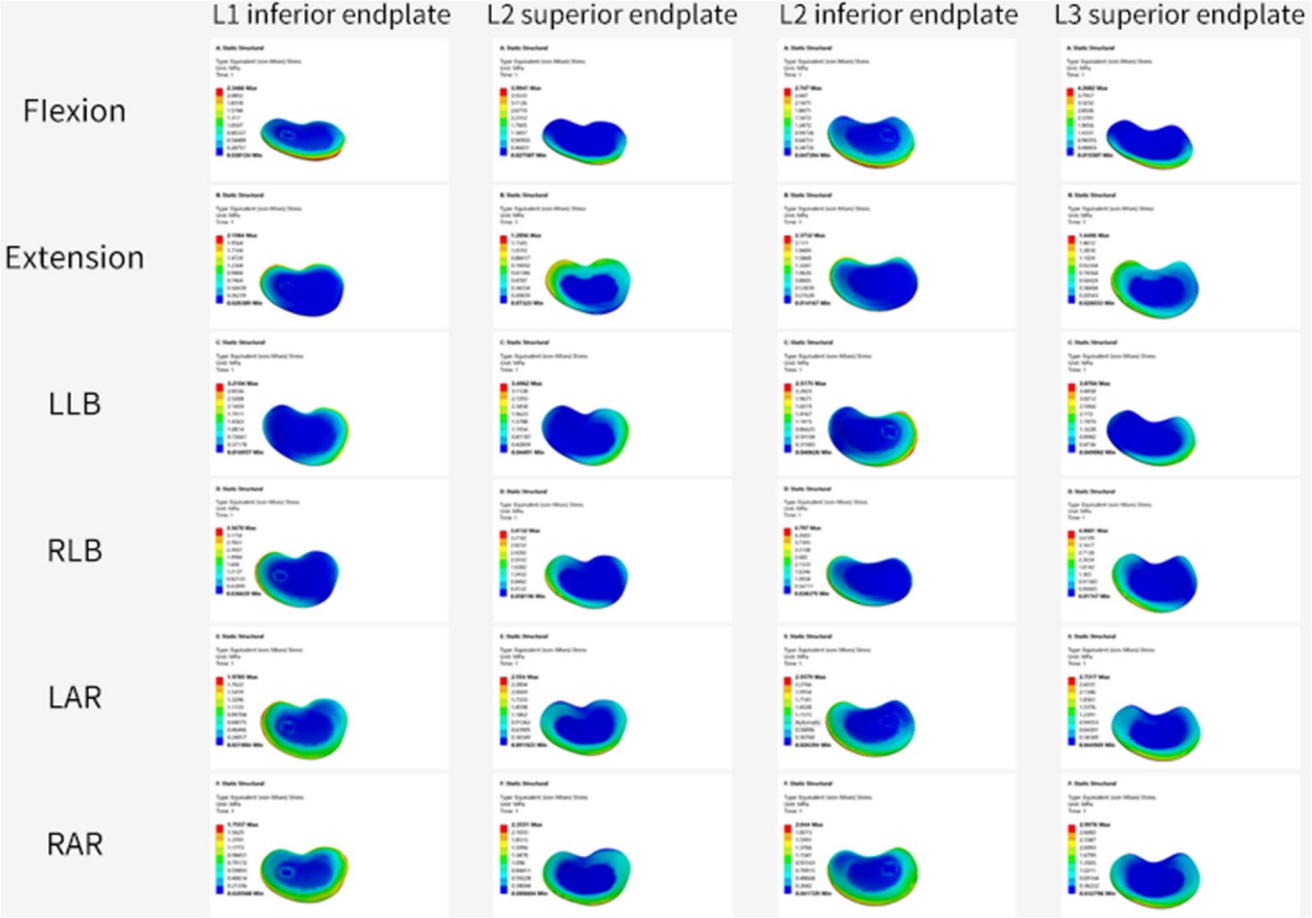
End plates stress cloud pictures of 4 ml bone cement in Bilateral integration group

**Fig. 7:**
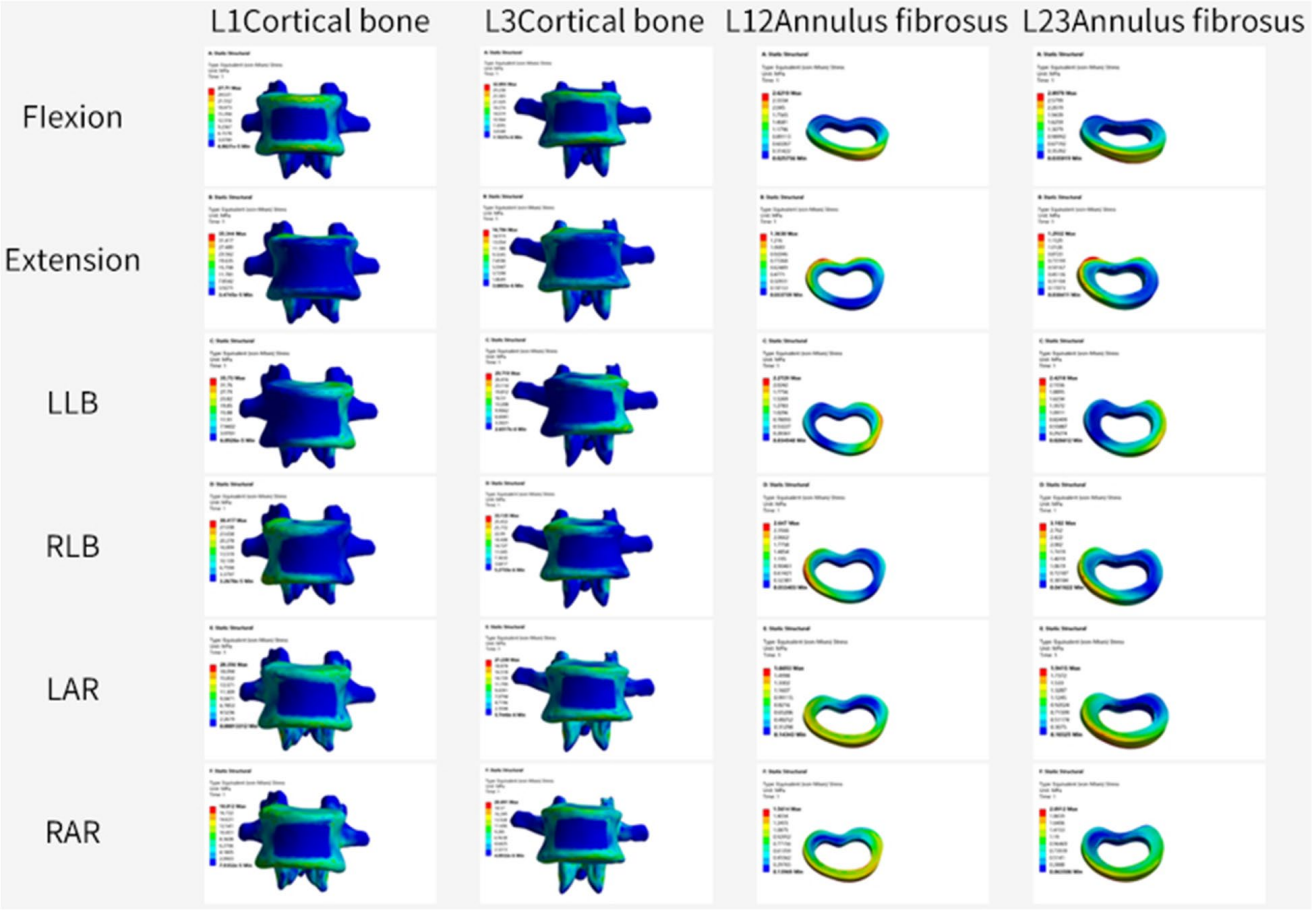
Vertebrae and annulus fibrosus stress cloud pictures of 4 ml bone cement in Bilateral integration group

We can find that no major difference between the number of milliliters of bone cement in the unilateral and bilateral separation groups from the above findings. In contrast in the BIG, when the cement injection volume was 4 ml, the maximum stress on the adjacent segment of the vertebral body was less than that of the unilateral and bilateral separation group. Although we obtained satisfactory results, the finite element analysis has some limitations after all, and further clinical validation may be required subsequently. Figures 6,7 show the pressure cloud when 4 ml of bone cement was injected in a double penetration one-body pattern, and we found that the stress area varied with the change in motion pattern. When the vertebral body moves in forward flexion and right side bending, the stresses on the adjacent structures are higher, while in left and right rotation, the stresses on the adjacent structures are lower.

## Discussion

Osteoporosis is a common disease in the elderly characterized by reduced bone strength, decreased bone mass, and a corresponding increase in fracture risk. Osteoporotic fractures have become a significant cause of disability and death in the elderly and seriously affect the quality of life of the elderly [24]. A large amount of literature reported [25–27], percutaneous kyphoplasty is effective in treating osteoporotic vertebral compression fractures, providing rapid pain relief, reducing bed rest, returning to normal life in the shortest possible time, improving quality of life, and reducing the incidence of complications caused by long-term bed rest and reduced activity in elderly patients. However, complications associated with it gradually emerge as the follow-up of patients increases. Tanigawa et al. [28]followed 194 patients for an average of 33 months. They found that 33% of patients had fractures after PKP, and 67% of fractures occurred in adjacent vertebrae, significantly higher than in non-adjacent segments. Secondary fractures in adjacent parts of vertebral body kyphoplasty are often the main complication, which seriously affects the long-term outcome and patient satisfaction after PKP. Therefore, in clinical treatment, how we choose the puncture method and the amount of bone cement injection that can reduce the stress on the adjacent segmental vertebral structures becomes a major problem for clinicians. Therefore, in this study, the finite element model was established and analyzed to find out the most suitable bone cement morphology and injection volume.

Since osteoporotic vertebral compression fractures mostly occur in the thoracolumbar segment, and the lumbar spine, as the main weight-bearing part of the spine, has a greatly increased risk of disease, the L1-L3 finite element model was established in this study with L2 as the study object. Compared with clinical studies and in vitro cadaveric experiments, the 3D finite element calculation method can reduce the experimental cost by defining material properties and simulating various actual conditions. In this experiment, the differences between different bone cement morphologies formed after percutaneous vertebroplasty and different bone cement injection volumes were simulated by establishing a bone mineralization model to explore the differences in the mechanical properties of adjacent structures in the L2 segment after bone cement filling. The present bone cement model uses the real intraoperative bone cement morphology, which is different from previous studies that simulated bone cement as cylindrical or spherical, and this method is more effective in simulating the real intraoperative bone cement morphology.

According to the present study, it was found that the three bone cement forms caused little difference in von Mises stress in the intervertebral structures when the bone cement injection volume was 2 ml. This is consistent with the finding of Liebschner et al. [29] that both bilateral pedicle and unilateral pedicle bone cement infusion restored vertebral body strength. However, several other studies have shown [30] that the efficacy of unilateral versus bilateral punctures is similar. Unilateral PKP is characterized by short operative time, low cost, and low cement usage. However, unilateral PKP shows a higher risk of adjacent vertebrae in fracture compared to bilateral PKP.

Several studies have been done on the effect of the morphology of bone cement in PKP surgery on the postoperative clinical outcome of OVCFs. In a study by Liu et al. [31], it was found that VAS, ODI scores, etc. were significantly improved at two days postoperatively and at the final follow-up by separated versus confluent bone cement compared to preoperative. The distribution of bone cement within the PKP in both groups was done in a manner that helped to relieve pain and reduce vertebral body biological curvature.In comparison between the two groups, the integration VAS score was better than the separated type on the second postoperative day, indicating that the integration distribution method has an advantage in short-term pain relief. This has some similarity with the present study, in which we found that when the bone cement injection volume was greater than 2 ml, the intervertebral structure was less stressed by the bilateral integration group bone cement form compared to the remaining two bone cement forms. This study reveals the superiority of the bilateral integration bone cement form from the mechanical point of view. However, in the study by He et al. [32], the "separated" cement distribution was better than the "integration" cement distribution at the one-year follow-up, suggesting that the "separated" distribution was better for analgesia. They concluded that the "separated" distribution method increases the contact area with the cancellous bone of the vertebral body and increases the "binding" function between the bone cement and the trabeculae. In addition, the "separated" distribution is closer to a plateau support than a "confluent" distribution with depressed support, which increases the stability of the vertebral body and reduces micromovements of the trabeculae, thus reducing residual back pain. Therefore, more in vitro trials and randomized controlled trials with large samples are needed to clarify the superiority of the bone cement distribution method.

The amount of bone cement injection during PKP surgery has been the focus of research by domestic and foreign scholars. In this experiment, it was found that the stresses on the intervertebral structures decreased as the injection volume of bone cement increased. Moreover, when 4 ml of bone cement was injected in the bilateral integration form, the stress on the adjacent segment was lower than the other two bone cement forms. Therefore, 4 ml is a more suitable amount of bone cement injection. This is consistent with the study of Wang et al. [33], who found 4 ml to be the optimal amount of bone cement injection after simulating fluid bone cement injection into the injured spine by building a three-dimensional finite element model of the thoracolumbar spine. More and more scholars are now paying more attention to the volume fraction of bone cement than the volume of bone cement injected, because it can more realistically reflect the relationship between the volume of bone cement and the volume of the fractured vertebrae, and thus more accurately guide the amount of bone cement used in the clinic. Kim's group reported [34] bone cement restores skeletal stiffness when the cement volume reaches 30% of the vertebral body volume; however, when the cement exceeds this volume, it leads to abnormal stiffness, which may increase spinal stresses. A study by Brojan M et al. [35] suggested that the amount of cement filling for vertebroplasty should be 15% of the volume of the fractured vertebral body, which corresponds to a cement filling of 4–6 ml. 15% is the limit of cement filling beyond which there is no significant increase in the stiffness of the vertebral body as well as in the pressure within the intervertebral disc. In contrast, Nieuwenhuijse et al. [36], calculated the cement infusion dose in 196 patients with OVCFs by CT examination after vertebroplasty and further explored the association between cement infusion dose and pain relief, showing that the average postoperative cement infusion dose was about 3.94 mL and it was the lowest threshold for their pain relief. Further studies showed better pain relief without increasing leakage and new fractures when the volume fraction of bone cement infused was 24%. This trial only focused on the most common milliliter of bone cement in clinical practice, and studies on the volume fraction of bone cement need to be supplemented and refined by subsequent trials.

In addition, this study found that when the vertebral body is flexion and lateral bending, the stress on the intervertebral structures is significantly greater than in other forms of motion, and conversely when the vertebral body is rotated left and right, the stress on the intervertebral structures is less. Therefore, during postoperative rehabilitation exercises, we recommend using left–right rotation as much as possible, while avoiding bending and lateral curvature to prevent fracture of the adjacent vertebrae.

There are certain limitations of our study and finite element model. There are different types of bone cement shapes due to the degree of dispersion of the bone cement within the vertebral body. In our study, the morphology of the original bone cement was simplified to facilitate calculations and analysis. The bone cement we analyzed was of the "agglomerate" type, which may have led to the overly homogeneous results of our tests. In addition to this, due to the diversity of vertebral morphology at the time of fracture, we did not model the fractured vertebrae and therefore could not reflect the complexity of the actual situation because several factors were not considered (such as the finite element model was overly simplistic, vertebral fracture conditions were not included in the study, and the effect of different cement distribution locations on the results was not taken into account). Still, it could provide data for subsequent fracture modeling support. All these reasons may influence the von Mises stresses observed after PKP surgery. Due to the inconsistent anatomical morphology of the nine models, there may be subtle variations in stresses from vertebra to vertebra, even when the same loading method is used. Based on the current study, future biomechanical analyses should include finite element models that reflect the human condition more accurately.

## Conclusions

The modeling results showed that the different bone cement injection methods and bone cement volume have substantial effect on the adjacent segmental vertebral structures. The bilateral integration bone cement produces approximately 10% less stress on adjacent segments than the remaining two cement forms and, when the injection volume is 4 ml, reduces stress on adjacent segments by approximately 15% while maintaining the stability of the injected vertebral body.

## Data Availability

All data are calculated by the software itself.The datasets used and/or analysed during the current study are available from the corresponding author on reasonable request.

## References

[1] Curtis EM, Moon RJ, Dennison EM, Harvey NC, Cooper C (2016). Recent advances in the pathogenesis and treatment of osteoporosis. Clin Med (Lond).

[2] Johnell O, Kanis JA (2006). An estimate of the worldwide prevalence and disability associated with osteoporotic fractures. Osteoporos Int.

[3] Suzuki N, Ogikubo O, Hansson T (2009). The prognosis for pain, disability, activities of daily living and quality of life after an acute osteoporotic vertebral body fracture: its relation to fracture level, type of fracture and grade of fracture deformation. Eur Spine J.

[4] Adachi JD, Ioannidis G, Olszynski WP, Brown JP, Hanley DA, Sebaldt RJ (2002). The impact of incident vertebral and non-vertebral fractures on health related quality of life in postmenopausal women. BMC Musculoskelet Disord.

[5] Chang W, Zhang X, Jiao N, Yuwen P, Zhu Y, Zhang F (2017). Unilateral versus bilateral percutaneous kyphoplasty for osteoporotic vertebral compression fractures: a meta-analysis. Medicine (Baltimore).

[6] Wang XR, Kwok TCY, Griffith JF, ManYu BW, Leung JCS, Wáng YXJ (2019). Prevalence of cervical spine degenerative changes in elderly population and its weak association with aging, neck pain, and osteoporosis. Ann Transl Med.

[7] Galibert P, Deramond H, Rosat P, Le Gars D (1987). Preliminary note on the treatment of vertebral angioma by percutaneous acrylic vertebroplasty. Neurochirurgie.

[8] Voormolen MH, Mali WP, Lohle PN, Fransen H, Lampmann LE, van der Graaf Y (2007). Percutaneous vertebroplasty compared with optimal pain medication treatment: short-term clinical outcome of patients with subacute or chronic painful osteoporotic vertebral compression fractures The VERTOS study. AJNR Am J Neuroradiol.

[9] Burton AW, Hamid B (2008). Kyphoplasty and vertebroplasty. Curr Pain Headache Rep.

[10] Garfin SR, Yuan HA, Reiley MA (2001). New technologies in spine: kyphoplasty and vertebroplasty for the treatment of painful osteoporotic compression fractures. Spine (Phila Pa 1976).

[11] Zhang L, Li J, Yang H, Luo Z, Zou J (2013). Histological evaluation of bone biopsy results during PVP or PKP of vertebral compression fractures. Oncol Lett.

[12] Ma X, Xing D, Ma J, Wang J, Chen Y, Xu W (2013). Risk factors for new vertebral compression fractures after percutaneous vertebroplasty: qualitative evidence synthesized from a systematic review. Spine (Phila Pa 1976).

[13] Zhang Z, Fan J, Ding Q, Wu M, Yin G (2013). Risk factors for new osteoporotic vertebral compression fractures after vertebroplasty: a systematic review and meta-analysis. J Spinal Disord Tech.

[14] Wilcox RK (2006). The biomechanical effect of vertebroplasty on the adjacent vertebral body: a finite element study. Proc Inst Mech Eng H.

[15] Baroud G, Nemes J, Ferguson SJ, Steffen T (2003). Material changes in osteoporotic human cancellous bone following infiltration with acrylic bone cement for a vertebral cement augmentation. Comput Methods Biomech Biomed Engin.

[16] Lu YM, Hutton WC, Gharpuray VM (1996). Can variations in intervertebral disc height affect the mechanical function of the disc?. Spine (Phila Pa 1976).

[17] Boger A, Heini P, Windolf M, Schneider E (2007). Adjacent vertebral failure after vertebroplasty: a biomechanical study of low-modulus PMMA cement. Eur Spine J.

[18] Han KS, Rohlmann A, Yang SJ, Kim BS, Lim TH (2011). Spinal muscles can create compressive follower loads in the lumbar spine in a neutral standing posture. Med Eng Phys.

[19] Peng Y, Du X, Huang L, Li J, Zhan R, Wang W (2018). Optimizing bone cement stiffness for vertebroplasty through biomechanical effects analysis based on patient-specific three-dimensional finite element modeling. Med Biol Eng Comput.

[20] Liang D, Ye LQ, Jiang XB, Yang P, Zhou GQ, Yao ZS (2015). Biomechanical effects of cement distribution in the fractured area on osteoporotic vertebral compression fractures: a three-dimensional finite element analysis. J Surg Res.

[21] Rohlmann A, Zander T, Rao M, Bergmann G (2009). Applying a follower load delivers realistic results for simulating standing. J Biomech.

[22] Li C, Zhou Y, Wang H, Liu J, Xiang L (2014). Treatment of unstable thoracolumbar fractures through short segment pedicle screw fixation techniques using pedicle fixation at the level of the fracture: a finite element analysis. PLoS One.

[23] Schmidt H, Heuer F, Simon U, Kettler A, Rohlmann A, Claes L (2006). Application of a new calibration method for a three-dimensional finite element model of a human lumbar annulus fibrosus. Clin Biomech (Bristol, Avon).

[24] Heary R, Bono C (2001). Metastatic spinal tumors. Neurosurgical focus.

[25] Cotten A, Boutry N, Cortet B, Assaker R, Demondion X, Leblond D (1998). Percutaneous vertebroplasty: state of the art*.* Radiographics : a review publication of the Radiological Society of North America. Inc.

[26] Deramond H, Depriester C, Galibert P, Le Gars D (1998). Percutaneous vertebroplasty with polymethylmethacrylate. Technique, indications, and results. Radiologic clinics of North America.

[27] Maynard A, Jensen M, Schweickert P, Marx W, Short J, Kallmes D (2000). Value of bone scan imaging in predicting pain relief from percutaneous vertebroplasty in osteoporotic vertebral fractures. AJNR American journal of neuroradiology.

[28] Tanigawa N, Kariya S, Komemushi A, Nakatani M, Yagi R, Kohzai M (2011). Percutaneous vertebroplasty for osteoporotic compression fractures: long-term evaluation of the technical and clinical outcomes. AJR Am J Roentgenol.

[29] Liebschner M, Rosenberg W, Keaveny T (2001). Effects of bone cement volume and distribution on vertebral stiffness after vertebroplasty. Spine.

[30] Tang J, Guo WC, Hu JF, Yu L (2019). Unilateral and bilateral percutaneous kyphoplasty for thoracolumbar osteoporotic compression fractures. J Coll Physicians Surg Pak.

[31] Liu H, Zhang J, Liang X, Qian Z, Zhou Z, Lu H (2019). Distribution Pattern Making Sense: Patients Achieve Rapider Pain Relief with Confluent Rather Than Separated Bilateral Cement in Percutaneous Kyphoplasty for Osteoporotic Vertebral Compression Fractures. World Neurosurg.

[32] He S, Zhang Y, Lv N, Wang S, Wang Y, Wu S (2019). The effect of bone cement distribution on clinical efficacy after percutaneous kyphoplasty for osteoporotic vertebral compression fractures. Medicine (Baltimore).

[33] Wang D, Li Y, Yin H, Li J, Qu J, Jiang M (2020). Three-dimensional finite element analysis of optimal distribution model of vertebroplasty. Ann Palliat Med.

[34] Kim JM, Shin DA, Byun DH, Kim HS, Kim S, Kim HI (2012). Effect of bone cement volume and stiffness on occurrences of adjacent vertebral fractures after vertebroplasty. J Korean Neurosurg Soc..

[35] Martinčič D, Brojan M, Kosel F, Štern D, Vrtovec T, Antolič V (2015). Minimum cement volume for vertebroplasty. Int Orthop.

[36] Nieuwenhuijse M J, Bollen L, van Erkel A R and Dijkstra P D, Optimal intravertebral cement volume in percutaneous vertebroplasty for painful osteoporotic vertebral compression fractures. Spine (Phila Pa 1976). 2012;37(20):1747–55. 10.1097/BRS.0b013e318254871c. 10.1097/BRS.0b013e318254871c 22433500

